# Evaluating the efficacy of a standardized 4 mL/kg fluid bolus technique in critically ill patients with elevated P_va_CO_2_: secondary analysis of two prospective studies

**DOI:** 10.3389/fmed.2024.1348747

**Published:** 2024-03-21

**Authors:** Rachid Attou, Thierry Du, Dimitrios Velissaris, Sebastien Redant, Mircea T. Talpoș, Charalampos Pierrakos

**Affiliations:** ^1^Department of Intensive Care, Brugmann University Hospital, Université Libre de Bruxelles, Brussels, Belgium; ^2^Department of Internal Medicine, University Hospital of Patras, Patras, Greece

**Keywords:** fluid challenge, PCO2 gap, carbon dioxide, veins/metabolism, plasma substitutes, crystalloid solutions, hemodilution, hemodynamics

## Abstract

**Background:**

Limiting the fluid bolus (FB) volume may attenuate side effects, including hemodilution and increased filling pressures, but it may also reduce hemodynamic responsiveness. The minimum volume to create hemodynamic effects is considered to be 4 mL/kg. In critically ill patients, the hemodynamic effects of FB with this volume have not been adequately investigated and compared to higher quantities. We hypothesized that a standardized FB approach using 4 mL/kg has comparable hemodynamic and metabolic effects to the common practice of physician-determined FB in critically ill patients.

**Methods:**

We conducted *post hoc* analysis of two trials in non-selected critically ill patients with central venous-to-arterial CO_2_ tension (P_va_CO_2_) >6 mmHg and no acute bleeding. All patients received crystalloids either at a physician-determined volume and rate or at 4 mL/kg pump-administered at 1.2 L/h. Cardiac index (CI) was calculated with transthoracic echocardiogram, and arterial and venous blood gas samples were assessed before and after FB. Endpoints were changes in CI and oxygen delivery (DO_2_) >15%.

**Results:**

A total of 47 patients were eligible for the study, 15 of whom received physician-determined FB and 32 of whom received standardized FB. Patients in the physician-determined FB group received 16 (12–19) mL/kg at a fluid rate of 1.5 (1.5–1.9) L/h, compared to 4.1 (3.7–4.4) mL/kg at a fluid rate of 1.2 (1.2–1.2) L/h (*p* < 0.01) in the standardized FB group. The difference in CI elevations between the two groups was not statistically significant (8.8% [−0.1–19.9%] vs. 8.4% [0.3–23.2%], *p* = 0.76). Compared to physician-determined FB, the standardized FB technique had similar probabilities of increasing CI or DO_2_ by >15% (odds ratios: 1.3 [95% CI: 0.37–5.18], *p* = 0.66 and 1.83 [95% CI: 0.49–7.85], *p* = 0.38).

**Conclusion:**

A standardized FB protocol (4 mL/kg at 1.2 L/h) effectively reduced the volume of fluid administered to critically ill patients without compromising hemodynamic or metabolic effects.

## Introduction

Fluid bolus (FB) is an essential technique used in the management of critically ill patients that involves the rapid administration of intravenous fluids under strict control and evaluating the patient’s hemodynamic response (1). The primary objectives of this technique are to increase cardiac output and, thus, oxygen delivery via the Frank–Starling law (2). The amount and rate of fluid infusion are essential components of this technique (1). Insufficient volume and rate may not result in a significant preload increase, leading to misleading results and suboptimal treatment of fatal hypovolemia (3, 4). On the other hand, increased volume can significantly increase cardiac pressures, possibly leading to pulmonary or peripheral edema, which can be detrimental for critically ill patients (5). Consequently, it is clinically significant to investigate the optimal fluid volume and rate for performing FB.

The optimal fluid volume and rate for a fluid challenge in critically ill patients has not yet been fully determined. In one study, an FB of approximately 1 L administered over 5–10 min significantly increased the cardiac index (CI) and filling pressures in healthy volunteers (6). According to standard practice, 500 mL of fluid is administered to critically ill patients at a rate of 1 L/h (7). Slower rates of FB have not been proven superior in terms of patients’ outcomes (8). Nonetheless, blood volume can vary according to body measurement. Consequently, a constant fluid dose for all patients may be insufficient or excessive for some patients. Prior research has shown that 4 mL/kg of crystalloid fluids is sufficient to increase mean systemic filling pressure and cause a significant elevation in CI in cardiac surgery patients (4). Nevertheless, the extent to which this finding may be applied to the wider population of critically ill individuals requiring FB to address insufficient CI for meeting their metabolic requirements has not been thoroughly examined.

A substantial increase in the difference between mixed venous and arterial CO_2_ partial pressure, particularly when it surpasses 6 mmHg, is a crucial marker of insufficient peripheral perfusion in clinical settings (9). This measure, obtained from Fick’s equation for CO_2_, is directly linked to the ratio of CO_2_ generation (VCO_2_) to cardiac output, assuming a wide but linear correlation between CO_2_ partial pressure and CO_2_ content within physiological limits (10). A high P_va_CO_2_ value indicates that the cardiac output is not enough to fulfill the metabolic needs, as seen by the inadequate removal of CO_2_ related to its production (11). In such instances in the context of critically ill patients, it becomes imperative to consider the administration of a FB in order to enhance cardiac output (11, 12). Hence, it is crucial to evaluate the efficacy of FB techniques in patients with high P_va_CO_2_ levels, since improper use of FB in these patients may result in less than ideal hemodynamic and/or metabolic results.

This study’s objective was to assess the hemodynamic and metabolic effects of the FB technique using a physician-determined volume and rate or a standardized volume of 4 mL/kg at a rate of 1.2 L/h. We hypothesized that in critically ill patients with limited peripheral perfusion, as indicated by elevated central venous-to-arterial CO_2_ tension (P_va_CO_2_), a standardized fluid challenge technique adapted to body weight could yield comparable hemodynamic outcomes to existing practices, but with a reduced volume of fluid administered.

## Methods

### Design and setting

In this *post hoc* analysis, we examined the data of patients enrolled in two prospective studies conducted at the 33-bed Intensive Care Unit (ICU) of Brugmann University Hospital in Brussels, Belgium, focusing on critically ill patients with P_va_CO_2_ levels greater than 6 mmHg who received FB. In the first cohort of patients, treated between January and June 2015, a physician-determined FB technique was applied, i.e., the rate and volume of fluid administration were at the attending physician’s discretion. The second cohort of patients, treated from September 2021 to March 2023, was managed under a standardized FB protocol (standardized technique; 1.2 L/h, 4 mL/kg per actual body weight). Both cohorts received FB with Plasma-Lyte A (Baxter Healthcare, Deerfield, IL) and were assessed for fluid responsiveness using transthoracic echocardiography. The methods and the results of the first trial have been previously described (13). The second cohort of patients represents a part of an ongoing prospective observational trial (ISRCTN58464956). Briefly, similar to the first study, this second study evaluated patients receiving Plasma-Lyte A (Baxter Healthcare, Deerfield, IL), assessing fluid responsiveness through transthoracic echocardiography and venous and arterial blood gas analysis. The objective of this ongoing trial is to explore the relationship between changes in P_va_CO_2_ and capillary refilling time during FB. The inclusion of this unpublished study is justified by its methodological consistency with the first study, while employing a different approach to FB, thus offering a unique opportunity to compare outcomes. Ethical approval for the study protocols (CE2014/122 and CE2021/29) was granted by the local ethics committee, and written informed consent was obtained from each patient or their next of kin.

### Inclusion and exclusion criteria

Patients with P_va_CO_2_ > 6 mmHg in whom the attending physician decided for an FB of crystalloids at any time of their stay in the ICU were considered eligible for this study. Each patient was assessed once.

The exclusion criteria were: (1) patients younger than 18 years old; (2) patients not equipped with jugular or subclavian venous catheter and arterial catheter; (3) measurement of cardiac output with cardiac ultrasound not possible due to lack of acoustic window; (4) patients receiving extracorporeal membrane oxygenation support; (5) partial pressure of CO_2_ (PCO_2_) exceeding 75 mmHg in venous or arterial blood gas analysis, as beyond this limit, the association between PCO_2_ and CO_2_ content may not be linear; (6) atrial fibrillation, to prevent any bias in the calculation of cardiac output through the evaluation of stroke volume using transthoracic echocardiography; (7) other simultaneous interventions (i.e., introduction or increase in inotrope dosage, previous FB, ventilator mode changes, or the introduction of mechanical ventilation) within 30 min before fluid administration; and (8) acute bleeding related to hemodynamic instability or need for transfusion of red blood cells.

### Data and sample collections

Demographics, the type of fluids used for FB, the reason for FB, concomitant treatments (mechanical ventilation, inotropic agents), and laboratory data were collected for each patient. The Acute Physiology and Chronic Health Evaluation (APACHE) II score upon admission was used to assess disease severity. Sepsis was defined according to the Sepsis-3 definition (14). Before and after FB, we performed arterial and central venous blood gas analyses (which were sampled simultaneously), including hemoglobin, arterial and venous oxygen pressure (P_a_O_2_ and P_v_O_2_, respectively), and oxygen saturation (S_a_O_2_ and S_cv_O_2_). Central venous pressure (CVP) was also evaluated.

The arterial and venous oxygen content (C_a_O_2_, C_v_O_2_), oxygen delivery (DO_2_), oxygen consumption (VO_2_), and oxygen extraction ratio were computed using validated formulas (15). Also, P_va_CO_2_ and its difference ratio with arterial–venous oxygen content (P_va_CO_2_/C_av_O2) were calculated as indicators of the adequacy of peripheral perfusion and of tissue hypoxia (16), respectively.

### Cardiac output calculation

An assessment of all patients was conducted using Doppler transthoracic echocardiography (GE Healthcare Vivid S5) both prior to and following the administration of FB. We determined the blood velocity time integral (VTI) of the left ventricular outflow tract (LVOT) prior to administering FB. In order to compute stroke volume and CI, the diameter of the LVOT was assessed in the parasternal long-axis view at the insertion sites of the aortic cusp., situated below the aortic valve. Immediately following FB, we repeated the measurements. Both measurements were archived for offline analysis. The mean velocity time integral was computed by averaging the values obtained from three consecutive velocity trajectories. We utilized the identical value of LVOT diameter to compute stroke volume and CI both prior to and following FB. Considering that the inter-examination least significant change in velocity time integral measurements is 11% (ranging from 5 to 18%) (17), we defined fluid responders as patients who exhibit an increase in cardiac index CI greater than 15% (18).

### Study endpoints

The study’s primary endpoint was an increase of >15% in CI and DO_2_. The total given volume and changes in P_va_CO_2_, S_cv_O_2_, lactate, and oxygen consumption were secondary endpoints.

### Analysis plan

There was no formal power analysis conducted for this exploratory study. Instead, all available patients in the trials served as the sample size for the analysis.

R was used to conduct statistical analyses via the R-studio interface (www.r-project.org, R version 3.3.1). For each variable analyzed, descriptive statistics were computed. Absolute changes (Δ = After FB value – Before FB value) and relative changes (d = [(After FB value – Before FB value) / Before FB value] × 100) of different variables were evaluated. Continuous variables were expressed as medians with interquartile ranges, and discrete variables as counts (percentages). Categorical variables were compared using Fisher’s exact test, and a Wilcoxon signed-rank test was used to compare continuous variables. A binomial logistic regression analysis was performed to determine the impact of FB on the probability of fluid responsiveness. We calculated odds ratios with 95% confidence intervals (95% CI). A *p* value <0.05 was employed to define statistical significance.

## Results

### Study population

Of the 47 patients who met the entry criteria and were analyzed, 15 underwent treatment with FB wherein the volume and rate were determined by the attending physician, while 32 were treated using a standardized FB technique. The group treated with the physician-determined FB received more fluids, averaging 16 (12–19) mL/kg compared to 4.1 (3.7–4.4) mL/kg in the standardized group (*p* < 0.01). They also received fluids at a faster rate, with an average of 1.5 (1.5–1.9) L/h versus 1.2 (1.2–1.2) L/h (*p* = 0.01) in the standardized group. Tables 1, 2 reveals no significant baseline differences between the two groups regarding disease severity, metabolic characteristics, or hemodynamic characteristics. Most patients in both groups were diagnosed with sepsis at the time of FB and received noradrenaline support during FB administration.

**Table 1:** Patient characteristics.

		Physician determined FB	Standardized FB	*p* values
Number of patients		15	32	
Time of examination after admission to ICU (days)		1 (1–2)	1 (1–5)	0.14
**Demographic characteristics**				
Age, years		76 (68–87)	72 (57–76)	0.08
Weight (kg)		60 (50–76)	75 (69–90)	0.01
Height (cm)		1.66 (1.61–1.72)	1.72 (1.61–1.81)	0.14
Medical admission type, *n* (%)		7 (46)	20 (62)	0.35
APACHE II score		22 (15–24)	24 (17–32)	0.98
Sepsis, *n* (%)		9 (60)	19 (59)	0.95
Shock, *n* (%)		7 (46)	12 (37)	0.45
Invasive ventilation, *n* (%)		8 (53)	16 (50)	0.86
	Tidal volume (ml/Kg^‡^)	7.2 (6.4–7.6)	7.4 (6.2–8.1)	
	PEEP (cmH_2_O)	6 (5–10)	10 (6–13)	
	P_a_O_2_/FiO_2_ (mm Hg)	177 (140–302)	180 (90–298)	
Lactate, mmol/L		2.1 (1.7–3.9)	2.2 (1.8–3.1)	0.75
Hemoglobin (mg/dL)		12.3 (9.8–13.8)	10.8 (8.9–11.5)	0.06
Fluid volume (mL/kg*)		16 (12–19)	4.1 (3.7–4.4)	<0.01
Fluid rate (L/h)		1.5 (1.5–1.9)	1.2	<0.01
Duration (min)		37 (30–40)	15 (15–20)	<0.01
**Principal reason for FB**				
	Hypotension, *n* (%)	4 (27)	12 (38)	0.52
	Hyperlactatemia, *n* (%)	8 (53)	15 (47)	0.76
	Oliguria or clinical signs of hypoperfusion, *n* (%)	3 (20)	5 (15)	0.69
Increase cardiac index >15%, *n* (%)		5 (33)	13 (40)	0.86

APACHE, Acute Physiology and Chronic Health Evaluation; PEEP, Positive end-expiratory pressure; P_a_O_2_, partial pressure of oxygen in arterial blood; FiO_2_, fraction of inspired oxygen; *: per actual body weight. ‡: predicted body weight.

**Table 2:** Metabolic and hemodynamic variables before fluid bolus (FB) according to physician-determined or standardized (4 mL/kg, 1.2 L/h) FB technique.

	Physician determined FB	Standardized FB	*p* values
Number of patients	15	32	
**Baseline metabolic variables**			
S_a_O_2,_ %	96 (93–97)	96 (92–98)	0.92
S_cv_O_2,_ %	58 (52–69)	62 (56–66)	0.71
P_va_CO_2,_ mmHg	10.1 (8.4–11.9)	8.1 (7.1–9.3)	0.06
P_va_CO_2_/C_av_O_2,_ mmHg/mL	1.8 (1.6–2.3)	1.8 (1.6–2.1)	0.96
Oxygen delivery, mL/min/m^2^	313 (207–472)	271 (311–336)	0.45
Oxygen consumption, mL/min/m^2^	95 (77–136)	89 (71–127)	0.35
Oxygen extraction, %	35 (29–45)	35 (28–39)	0.99
**Baseline hemodynamic variables**			
Cardiac index, L/min/m^2^	1.8 (1.4–2.6)	1.9 (1.4–2.7)	0.89
Stroke volume, mL	46 (31–62)	46 (34–56)	0.78
Heart rate, beats/min	94 (77–106)	89 (73–112)	0.88
Pulse pressure, mmHg	52 (48–61)	63 (53–75)	0.06
Mean arterial pressure, mmHg	82 (74–86)	71 (63–71)	0.12
Central venous pressure, mmHg	8 (3–12)	7 (1–11)	0.43

S_a_O_2_, arterial oxygen saturation; S_cv_O_2_, central venous oxygen saturation; P_va_CO_2_, venous-to-arterial carbon dioxide partial pressure difference; C_av_O_2_, arterial-to-venous oxygen content difference.

### Relation between fluid challenge technique and cardiac index changes

**Figure 1:**
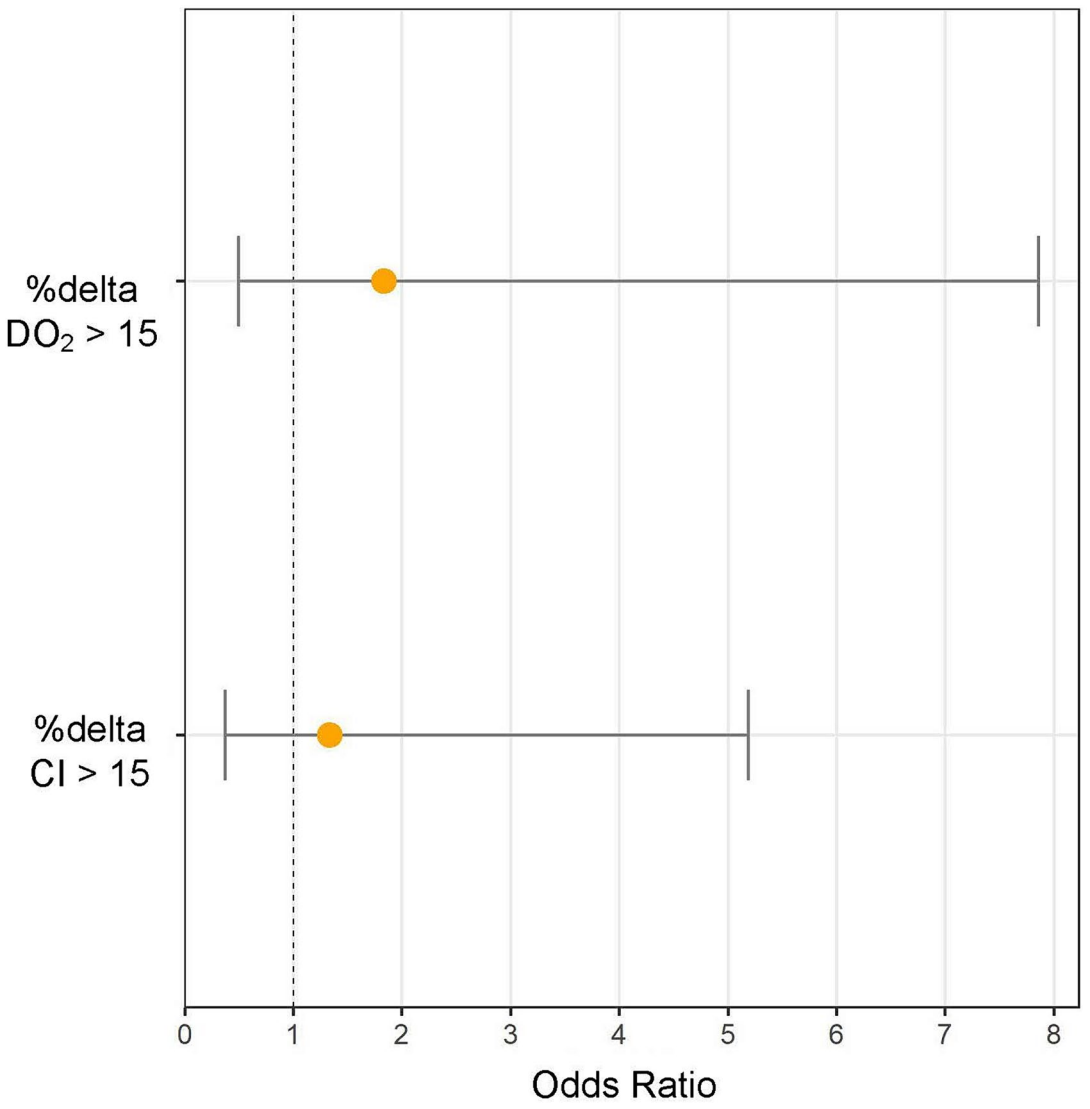
Increasing cardiac index (%Δ CI) and oxygen delivery (%Δ DO_2_) >15% odds ratios (and 95% CI) for standardized (4 mL/kg, 1.2 L/h) fluid bolus (FB) technique extracted from the multivariable logistic regression analysis with physician-determined FB as reference group.

Patients in the standardized FB group (receiving 4 mL/kg) did not demonstrate a lower likelihood of experiencing a > 15% increase in CI compared to those treated with the physician-determined FB technique (odds ratio: 1.3, 95% CI: 0.37–5.18, *p* = 0.66) (Figure 1). The standardized technique resulted in a similar increase in CI to the physician-determined technique (8.4% [0.3–23.2%] vs. 8.8% [−0.1–19.9%], *p* = 0.76) despite a lower increase in CVP (2 [0–3] mmHg vs. 4 (2−6) mmHg, *p* = 0.01) (Table 3).

**Table 3:** Hemodynamic changes after fluid bolus (FB) according to physician-determined or standardized (4 mL/kg, 1.2 L/h) FB technique.

	Physician determined FB	Standardized FB	*p* values
Number of patients	15	32	
**Hemodynamic variables changes**			
d cardiac index (%)	8.8 (−0.1–19.9)	8.4 (0.3–23.2)	0.76
Δ cardiac index (L/min/m^2^)	0.24 (−0.01–0.49)	0.28 (0.01–0.43)	0.99
d stroke volume (%)	12.4 (−2.5–21.6)	11.1 (2.7–22.2)	0.93
Δ stroke volume (mL)	5.1 (−0.7–10.9)	5.9 (0.7–10.2)	0.85
d heart rate (%)	−1.3 (−5.6–2.5)	0.0 (−5.9–1.5)	0.91
Δ heart rate (beats/min)	−1.0 (−4.1–1.8)	0.0 (−5.3–1.1)	0.91
d pulse pressure (%)	6.2 (−6.1–25.9)	5.1 (−5.9–14.6)	0.43
Δ pulse pressure (mmHg)	3 (−3–13)	3 (−3–11)	0.66
d mean arterial pressure (mmHg)	1.6 (−3.8–10.6)	2.5 (0.4–9.2)	0.51
Δ mean arterial pressure (mmHg)	1 (−4–8)	2 (−1–8)	0.42
d central venous pressure (%)	33 (30–71)	27 (0–51)	0.21
Δ central venous pressure (mmHg)	4 (2–6)	2 (0–3)	0.01

d, delta; S_cv_O_2_, central venous oxygen saturation; P_va_CO_2_, venous-to-arterial carbon dioxide difference; C_av_O_2_, arterial–venous oxygen difference ratio.

### Relation between fluid challenge technique and oxygen delivery changes

**Figure 2:**
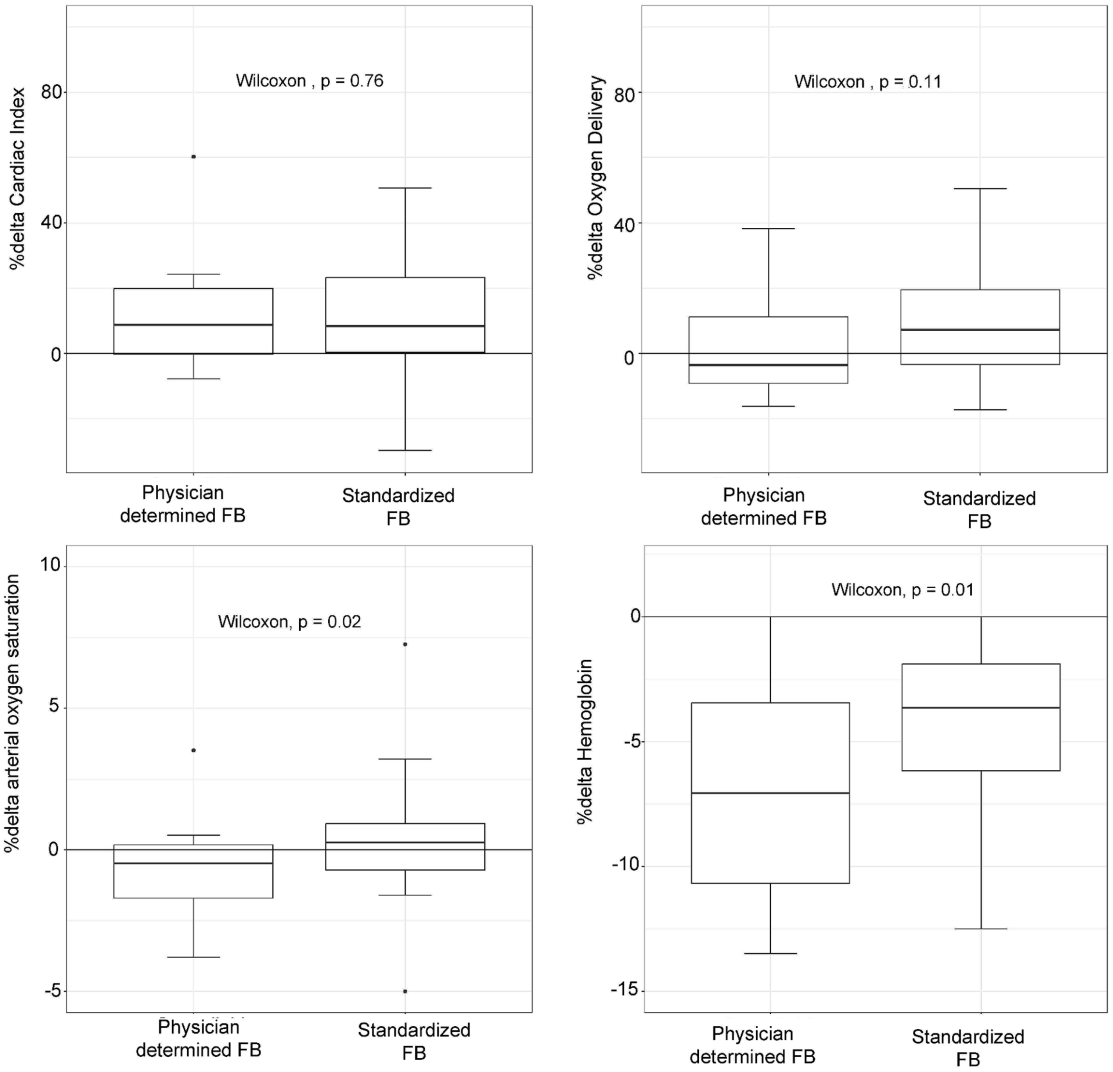
Oxygen delivery, cardiac index, hemoglobin concentration, and arterial oxygen saturation changes (%Δ) according to physician-determined or standardized (4 mL/kg, 1.2 L/h) fluid bolus (FB) technique.

Patients in the standardized FB group did not show a lower likelihood of a > 15% increase in DO_2_ compared to the physician-determined FB group (odds ratio: 1.83, 95% CI: 0.49–7.85, *p* = 0.38) (Figure 1). Although not statistically significant, the standardized FB technique resulted in a higher increase in DO_2_ compared to the physician-determined technique (7.2% [−3.8–19.3%] vs. -3.5% [−9.1–11.2%], *p* = 0.11) (Figure 2). Additionally, the standardized technique led to a smaller decrease in hemoglobin levels (−3.6% [−6.1−−1.8%] vs. -7.5% [−11.5−−3.6%], *p* = 0.01). A statistically significant decrease in oxygen saturation was observed with the physician-determined FB technique (−0.5% [−1.8–0.15%]) compared to an increase with the standardized FB technique (0.26% [−0.71–0.92%]) (*p* = 0.02) (Figure 2).

### Relation between fluid challenge technique and metabolic parameters changes

**Figure 3:**
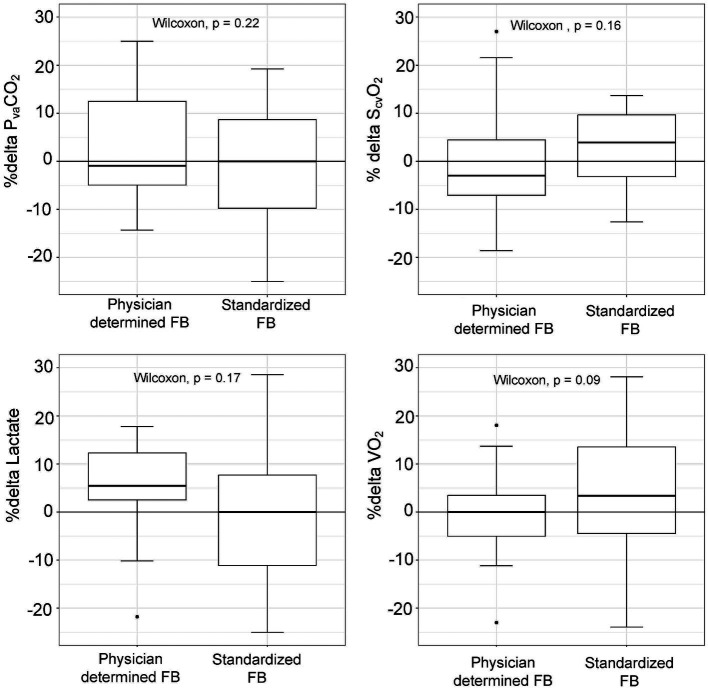
Changes in metabolic parameters (%delta) according to physician-determined or standardized (4 mL/kg, 1.2 L/h) fluid bolus (FB) technique.

There were no statistically significant differences in baseline P_va_CO_2_ values between patients treated with the standardized or physician-determined FB techniques (8.1 [7.1–9.3] mmHg vs. 10.1 [8.4–11.9] mmHg, *p* = 0.06) (Table 2). Both techniques resulted in comparable changes in P_va_CO_2_ (−5.6% [−22–10%] for the standardized technique vs. -19.7% [−39–0.3%] for the physician-determined technique, *p* = 0.22) (Figure 3). Additionally, no significant variations in S_cv_O_2_, lactate, or oxygen consumption values were observed between the two groups (Figure 3).

## Discussion

The study’s findings can be summed up as follows: (1) by employing a standardized FB technique, the volume of FB administered in relation to body weight was significantly reduced; (2) compared to those treated with higher volumes, critically ill patients who received crystalloids at a standardized dosage of 4 mL/kg not only exhibited a comparable likelihood of experiencing >15% increases in DO_2_ and CI, but also underwent metabolic effects comparable to those observed with a 16 mL/kg FB dosage; and (3) the standardized FB technique mitigated hemoglobin level reductions and reduced the risk of oxygen saturation decline.

The clinical significance of our study is highlighted by the existing variability in FB techniques in critical care practices (7). The type of fluids, volume, and rate are major components of the fluid challenge technique (1). In this study, we examined whether adhering to strict limits in these components could be effective under the pragmatic conditions of intensive care, demonstrating the hemodynamic and metabolic effectiveness of this approach. Notably, our findings indicate that patients who do not show improved hemodynamic or metabolic parameters after the initial standardized FB are unlikely to benefit from a repeated FB with a larger volume. This finding challenges the prevailing belief that higher fluid volumes are more efficacious, a concept our trial did not support (2). Our study demonstrates that a 4 mL/kg fluid bolus (FB) with a rate of 1.2 L/h of crystalloids can effectively assess fluid responsiveness and address hemodynamic and metabolic deficits, thus introducing a comprehensive and structured approach to FB in a field where standardization is lacking. While further research is required to determine if this standardized method outperforms physician-determined FB techniques in improving patient outcomes, our findings significantly address a critical gap in fluid resuscitation practices.

This study’s findings are consistent with previous research indicating that a FB of 4 mL/kg may have significant hemodynamic effects in post-cardiac surgery patients (4) and elective neurosurgical patients (19). We used the same amount of volume in non-selected critically ill patients. Almost half of those who received 4 mL/kg in our study experienced an increase in CI greater than 15%, comparable to the proportion of fluid responders in previous studies (20). The amount of fluid administered under a non-standardized technique resulted in a greater volume proportional to the patient’s weight but did not produce more pronounced hemodynamic effects. Therefore, the results of our study contribute to our comprehension of the optimal volume for FB in critically ill patients, suggesting that FB’s hemodynamic effects are no longer volume dependent for volumes greater than 4 mL/kg.

In the physician-determined FB technique, a higher fluid rate was used compared to the standardized FB, yet this did not result in a markedly stronger hemodynamic response. This finding contrasts with previous studies that associated fluid responsiveness with the rate of FB administration (3, 19). This disparity may stem from our study’s emphasis on the rate of fluid administration rather than its duration. In the physician-determined FB method, the duration surpassed 30 min, while in the standard approach, it was less than 20 min, despite a slower fluid rate. Moreover, the standardized technique employed a pump to ensure even fluid distribution throughout the FB process. Most patients were evaluated within 24 h of encountering a critical illness, a time often characterized by significant endothelial glycocalyx damage and increased vascular permeability (21). Given the short half-life of fluids in critically ill patients, approximately 15 min (22), the timing of assessing fluid responsiveness becomes pivotal. In the physician-determined FB group, the longer duration allowed for a substantial portion of the fluid to transition from the intravascular to the extravascular spaces. Additionally, the higher initial rate in this method may have exacerbated fluid leakage (23). Notably, some patients in the physician determined FB showed only minimal changes in hemoglobin levels after the FB, suggesting a modest expansion in intravascular volume. This likely attenuated the observable hemodynamic effects, explaining why a higher fluid rate did not lead to more pronounced results compared to the standardized FB technique. Therefore, this study sheds light on the hemodynamic response to FB at a commonly used in clinical practice rate of 1.2 L/h (7, 8), suggesting that higher rates may not significantly enhance this response.

In this study, we examined the effects of a standardized FB technique on DO_2_, alongside other metabolic parameters. While the primary objective of FB is to enhance CI and thereby increase DO_2_ (2), achieving this in practice can be challenging due to the unavoidable hemodilution effect of FB (24). Yet in clinical practice, it is essential to consider additional parameters like S_cv_O_2_ and blood lactate levels when assessing the adequacy of DO_2_ (25). Therefore, our evaluation included these metabolic markers in conjunction with DO_2_. Echoing findings from previous studies, we observed that reducing the volume of FB could mitigate the decrease in hemoglobin concentration (26). When comparing FB doses of 4 mL/kg and approximately 16 mL/kg, we noted a smaller decline in hemoglobin concentration at the lower dosage. This was correlated with a trend toward a higher increase in DO_2_ and S_cv_O_2_, as well as a more notable decrease in lactate levels. However, it is important to note that these differences did not achieve statistical significance. Therefore, our analysis reveals that while administering a 4 mL/kg volume of crystalloids can mitigate some hemodilution effects of FB, hemodilution remains considerable, leading to only subtle increases DO_2_ in several patients. This finding underscores the necessity of a multifaceted approach when evaluating the efficacy of FB in clinical settings, taking into account both hemodynamic and metabolic parameters.

In addition to hemodilution, significant adverse effects that may result from fluid administration regardless of the response to FB, such as deterioration of respiratory function, must be thoroughly investigated (5). CVP has been proposed as a safety parameter for patients receiving FB treatment (27). In our study, FB increased CVP in both groups. Nonetheless, FB with physician-determined volume and fluid rate caused a significant increase in CVP, reaching up to 6 mmHg in some patients. In contrast, patients receiving 4 mL/kg of FB exhibited modest increases in CVP, not exceeding 3 mmHg. Importantly, in this group of patients, oxygen saturation decreased less frequently than in patients who underwent the physician-determined FB technique. Therefore, the results of this investigation suggest that volume expansion with 4 mL/kg does not likely impair respiratory function by increasing cardiac filling pressure. Further evaluation is required to determine whether a 3-mmHg increase in CVP can be used as a safety limit in FB.

This study’s greatest asset is its comprehensive evaluation and comparison of FB techniques within a diverse group of critically ill patients, including a notable proportion with sepsis. Most of these patients were assessed early after admission. Hence, the study’s findings are particularly applicable to critically ill patients in the optimization and stabilization phase of their treatment, as well as during the de-escalation phase of fluid therapy (28). The uniformly elevated P_va_CO_2_ levels across all patients in both cohorts signal a suboptimal pre-FB CI, a conclusion further reinforced by the high blood lactate levels observed in most of these patients. Consequently, FB emerges as a potentially effective intervention for patients in both cohorts. Furthermore, a consistent frequency of true fluid responders across both groups is anticipated, with minimal differences in the potential for hemodynamic and metabolic improvements. This uniformity is vital, especially considering our study’s primary goal of validating or refuting the equivalence of different FB techniques by minimizing the risk of Type II statistical error.

Our study has limitations that merit consideration. First, no formal sample size calculation was conducted, and the small sample size, albeit comparable to prior studies (4, 19), may affect the robustness of our findings. However, the observed benefits in CI and DO_2_ with the 4 mL/kg FB technique suggest that a larger sample is unlikely to contradict these results. As a secondary analysis of two prospective trials, potential biases from our retrospective approach and non-preplanned hypotheses, especially due to unmeasured confounders, need to be acknowledged. Our specific exclusion criteria, such as the absence of an acoustic window for cardiac ultrasound or the presence of atrial fibrillation, could limit the generalizability of our findings. The study’s comparability may be compromised by the historical control bias arising from different treatment periods (2015 vs. 2021–2023) and by the variability in treatment application, with the first cohort’s fluid bolus technique based on physician discretion and the second cohort following a standardized protocol, potentially confounding the outcomes. Another significant limitation is our omission of post-FB microcirculation alterations assessment. Additionally, the lack of systematic evaluation of septic patients within the first hour of sepsis diagnosis (29) may impact the findings’ applicability to early sepsis management. The study also did not investigate the shock state of patients or the duration of hemodynamic effects post-FB. The study inclusion criteria necessitated patient stability, as indicated by stable inotrope levels for 30 min prior to evaluations, which could further restrict the generalizability of our results in the early phase of rescue fluid therapy (28). Furthermore, our threshold of a 15% increase in CI for fluid responders may miss clinically significant but smaller CI increases due to the limitations of transthoracic echocardiography in detecting subtle changes, potentially underestimating the number of true fluid responders.

## Conclusion

In a diverse cohort of critically ill patients, our study demonstrates that a standardized fluid bolus technique of 4 mL/kg at 1.2 L/h shows similar increases in cardiac index and oxygen delivery compared to techniques using larger volumes, with the additional benefit of reduced hemodilution and elevations in cardiac filling pressures. This balance of efficacy and safety suggests potential advantages in using this technique for fluid resuscitation in critical care scenarios.

## Data availability statement

The original contributions presented in the study are included in the article/supplementary material, further inquiries can be directed to the corresponding author.

## Ethics statement

The studies involving humans were approved by CHU-Brugmann ethical committee, Place Arthur Van Gehuchten 4 1020 Brussels (Laeken), Belgium. The studies were conducted in accordance with the local legislation and institutional requirements. The participants provided their written informed consent to participate in this study.

## Author contributions

RA: Data curation, Supervision, Validation, Writing – review & editing. TD: Conceptualization, Validation, Visualization, Writing – review & editing. DV: Supervision, Validation, Visualization, Writing – review & editing. SR: Supervision, Validation, Visualization, Writing – review & editing. MT: Supervision, Validation, Visualization, Writing – review & editing. CP: Conceptualization, Data curation, Formal analysis, Investigation, Methodology, Supervision, Validation, Visualization, Writing – original draft, Writing – review & editing.
